# Dynamic Behavior Analysis and Stability Control of Tethered Satellite Formation Deployment

**DOI:** 10.3390/s22010062

**Published:** 2021-12-23

**Authors:** Kangyu Zhang, Kuan Lu, Xiaohui Gu, Chao Fu, Shibo Zhao

**Affiliations:** 1 Institute of Vibration Engineering, Northwestern Polytechnical University, Xi’an 710072, China; zhangkangyu@mail.nwpu.edu.cn (K.Z.); fuchao0606@mail.nwpu.edu.cn (C.F.); zhaoshibo@mail.nwpu.edu.cn (S.Z.); 2 State Key Laboratory Mechanical Behavior and System Safety of Traffic Engineering Structures, Shijiazhuang Tiedao University, Shijiazhuang 050043, China; guxh@stdu.edu.cn

**Keywords:** tethered satellite formation, dynamic behavior, control, stable deployment, Floquet theory

## Abstract

In recent years, Tethered Space Systems (TSSs) have received significant attention in aerospace research as a result of their significant advantages: dexterousness, long life cycles and fuel-less engines. However, configurational conversion processes of tethered satellite formation systems in a complex space environment are essentially unstable. Due to their structural peculiarities and the special environment in outer space, TSS vibrations are easily produced. These types of vibrations are extremely harmful to spacecraft. Hence, the nonlinear dynamic behavior of systems based on a simplified rigid-rod tether model is analyzed in this paper. Two stability control laws for tether release rate and tether tension are proposed in order to control tether length variation. In addition, periodic stability of time-varying control systems after deployment is analyzed by using Floquet theory, and small parameter domains of systems in asymptotically stable states are obtained. Numerical simulations show that proposed tether tension controls can suppress in-plane and out-of-plane librations of rigid tethered satellites, while spacecraft and tether stability control goals can be achieved. Most importantly, this paper provides tether release rate and tether tension control laws for suppressing wide-ranging TSS vibrations that are valuable for improving TSS attitude control accuracy and performance, specifically for TSSs that are operating in low-eccentricity orbits.

## 1. Introduction

In recent years, satellite development has rapidly increased worldwide [1,2]. In particular, Tethered Space Systems (TSSs) have received significant attention in aerospace research as a result of their significant advantages: dexterousness, long life cycles and fuel-less engines [3,4,5]. TSSs are a new class of space vehicle that join two or more spacecraft together into a single structure by using soft tethers [6,7]. TSSs are utilized in man-made microgravity environments [8,9], spacecraft orbit transfers [10,11] and space debris cleanup [12,13], and they exhibit stronger reliability, higher stability and more diversified functions [14,15] when compared to traditional satellites. Tethered satellites are in unstable states during deployment without effective control as a result of disturbances produced by the space environment [16,17]. Due to their structural peculiarities, gravitational forces, aerodynamic drag, solar radiation pressure and other disturbances produced by the special environment in outer space, TSS vibrations are easily produced. These types of vibrations are extremely harmful to spacecraft.

TSS dynamics and control aspects have received considerable attention in recent decades [18,19,20]. Rigid-rod tether models provide analytical solutions of TSS and are widely used in basic research. For example, Williams [21] proposed a new feedback control scheme in which electrodynamic tether vibrations were suppressed, and tether stability was effectively controlled during deployment by using only electric current modulations. In another study, interorbital rendezvous with small relative inclination was also analyzed, and a nonlinear receding horizon controller was considered for tracking highly nonlinear systems by producing disturbances in system mass distributions and perturbations to initial system conditions [22]. Pradeep and Kumar [23] proposed nonlinear feedback tension control laws based on Liapunov’s method, used a linear state variable feedback control and affirmed the desired length of extended tethers in a reasonable amount of time.

Stability analysis is a core research focus in mechanism studies of dynamic systems. The Floquet theory is a stability theory of solutions of linear ordinary differential equations with periodic variable coefficients [24] that was proposed by G. Floquet in 1868. Few researchers used the Floquent theory to study TSS stability and dynamic behavior. Yu et al. [25] analyzed the spinning stability of a three-body Tethered Satellite Formation (TSF) by using Floquet theory, and stability analysis indicated that unstable motion occurs if its spinning angular rate is less than the critical value |−2.8| or 0.65 times its orbital angular rate. In another study, an analytical tether length rate control was designed, and parameter regions for stable deployment in order to maintain a tensile tether state were obtained [26]. Ellis and Hall [27] analyzed the stability of out-of-plane vibrations of a spinning TSS, and two satellites as point masses were connected by a rigid rod, constraining the system’s mass center to a circular orbit.

The aforementioned references indicate that little attention has been given to numerical studies on the accuracy of simplified TSS models, control stability and the influence of orbital eccentricity. However, tethers produce in-plane and out-of-plane swings and longitudinal and transverse vibrations as a result of complex perturbations [28,29], and a slight change in orbital eccentricity can significantly affect the original system. Previous studies show that analytical solutions for complex nonlinear models of TSS are difficult to obtain. This paper evaluates the stability of periodic TSS motions by using Floquet theory and provides control laws and small parameter domains of stability based on a simplified rigid-rod tether model.

In this paper, nonlinear dynamic behavior and stability of TSS during deployment are analyzed. A simplified rigid-rod model of a two-body tethered satellite is described in Section 2. Two simplified models of three-DOF equations are discussed in Section 3. Two control laws of tether release rate and tether tension are proposed in Section 4. The periodic stability of time-varying control systems is analyzed by using Floquet theory in Section 5. Conclusions are discussed in Section 6.

## 2. TSS Equations of Motion Using Lagrangian Method

**Figure 1:**
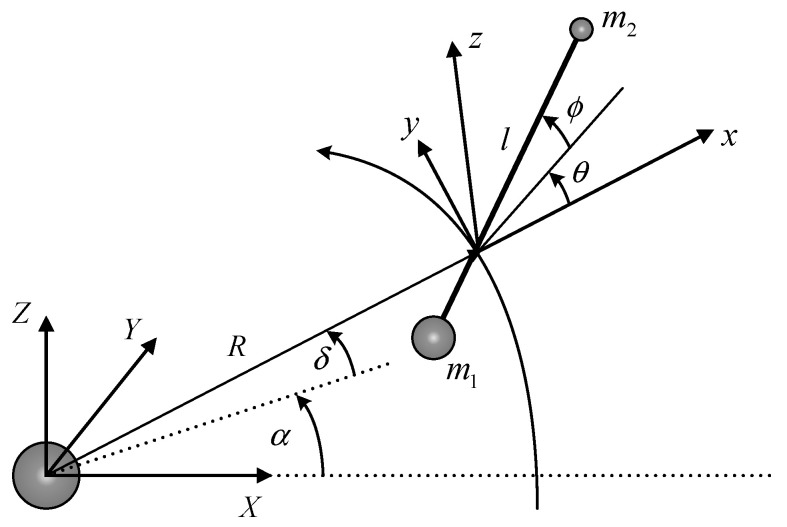
Rigid tether dynamical model.

Since the 1990s, TSS theory has developed rapidly from rigid-rod models to bead models and from continuous models to discrete models, and model accuracy is constantly improving [30,31]. However, other models’ dynamics equations are more complex and the quantity of computation is larger when compared with rigid-rod models [32]. The classical rigid-rod model is widely used. As shown in Figure 1, a rigid tethered satellite system consists of a mother satellite, $m_{1}$, and a subsatellite, $m_{2}$, (mass points), respectively. Both satellites are connected by a rigid tether. In this model, regardless of tether flexibility and tether elasticity, tethers released into outer space are considered as straight rods of infinite stiffness that do not bend or twist. Tether wounds on a spool of a deployment device in the mother satellite and tether length can be effectively controlled by the device.

The inertial geocentric frame, O−XYZ, the orbital frame, o−xyz, and the tether body frame, $o-x_{b}y_{b}z_{b}$, are all radial–transversal out-of-plane frames, and these are established in order to describe TSS position and attitude in Figure 1. Orbital radius and tether length are expressed as R, l, respectively; right ascension and declination to the center of mass are expressed as α, δ, respectively; and the tether in-plane angle and tether out-of-plane angle are expressed as θ, ϕ, respectively.

The radius vector with respect to the center of mass is written in inertial coordinates as follows.

(1) R=Rcosδcosαi+Rcosδsinαj+Rsinδk.

Total kinetic energy, $T_{k}$, consists of the translation of the center of mass, $T_{t}$, system rotation, $T_{r}$, and tether deployment, $T_{e}$:

(2) $$T_{t}=\frac{1}{2}m\left({\dot{R}}^{2}+R^{2}{\dot{\delta }}^{2}+R^{2}{\dot{\alpha }}^{2}\cos^{2}\delta \right)$$

where $m=m_{1}+m_{2}+m_{t}$ is the total system mass, $m_{t}=\rho l=m_{1}^{0}-m_{0}$ is the tether mass released into the external environment, ρ is the tether linear density and $m_{1}^{0}$ is the mass of the mother satellite before tether deployment that includes the tether mass:

(3) $$T_{r}=\frac{1}{2}{\left\{\omega \right\}}^{T}[I]\{\omega \}$$

where I is the tensor matrix of moment of inertia of the tethered satellite system, and ω is the inertial angular velocity of the tether in the inertial frame [33], which can be expressed as follows:

(4) $$\begin{array}{l} \omega =(\dot{\alpha }\sin\delta \cos\theta \cos\phi +\dot{\theta }\sin\phi -\dot{\delta }\sin\theta \cos\phi +\dot{\alpha }\cos\delta \sin\phi )i\\ \;\;\;\;\;\;\;-(\dot{\phi }+\dot{\alpha }\sin\delta \sin\theta +\dot{\delta }\cos\theta )j\\ \;\;\;\;\;\;\;+(\dot{\theta }\cos\phi -\dot{\alpha }\sin\delta \cos\theta \sin\phi +\dot{\delta }\sin\theta \sin\phi +\dot{\alpha }\cos\delta \cos\phi )k \end{array}$$

(5) $$\begin{array}{l} T_{r}=\frac{1}{2}m*l^{2}[{\left(\dot{\phi }+\dot{\alpha }\sin\delta \sin\theta +\dot{\delta }\cos\theta \right)}^{2}\\ \;\;\;\;\;\;+{\left(\dot{\delta }\sin\theta \sin\phi -\dot{\alpha }\sin\delta \cos\theta \sin\phi +\dot{\alpha }\cos\delta \cos\phi +\dot{\theta }\cos\phi \right)}^{2}] \end{array}$$

where $m*={(m}_{1}{+m}_{t}{/2)(m}_{2}{+m}_{t}/2)/m-m_{t}/6$ is the reduced mass of the system.

(6) $$T_{e}=\frac{1}{2}\frac{m_{1}(m_{2}+m_{t})}{m}{\dot{l}}^{2}.$$

The tether is assumed to be stationary relative to the mother satellite within the winch control mechanism, and its speed is provided during deployment.

When TSS systems are active in space, they are still within Earth’s gravitational field, and their potential energy is caused by Earth’s attraction. Potential energy is obtained from the mother satellite, the subsatellite and the tether, which is simplified by taking the first term of Maclaurin’s series expansion. We assume that a spherical earth is considered as follows:

(7) $$V=-\frac{\mu_{e}m}{R}+\frac{\mu_{e}m*l^{2}}{2R^{3}}(1-3\cos^{2}\phi \cos^{2}\theta )$$

where $\mu_{e}=398,600\;{\mathrm{km}}^{3}{/s}^{2}$ is Earth’s gravitational coefficient. The Lagrange function can be formed as follows.

(8) $$L=T_{t}+T_{r}+T_{e}-V.$$

By substituting Equation (8) into Lagrange’s equations and by assuming that the system’s center of mass is running in a constant orbital plane (δ=0), the system’s equations of motion can be obtained as follows:

(9) $$m\ddot{R}-mR{\dot{\alpha }}^{2}+\frac{\mu m}{R^{2}}-\frac{3\mu m*l^{2}}{2R^{4}}(1-3\cos^{2}\theta \cos^{2}\phi )=Q_{R}$$

(10) $$\begin{array}{l} 2mR\dot{R}\dot{\alpha }+\dot{m}*l^{2}(\dot{\alpha }+\dot{\theta })\cos^{2}\phi +2m*l\dot{l}[(\dot{\alpha }+\dot{\theta })\cos^{2}\phi ]\\ +m*l^{2}[(\ddot{\alpha }+\ddot{\theta })\cos^{2}\phi -2(\dot{\alpha }+\dot{\theta })\dot{\phi }\sin\phi \cos\phi ]+mR^{2}\ddot{\alpha }=Q_{\alpha } \end{array}$$

(11) $$\begin{array}{l} m*l^{2}[(\ddot{\alpha }+\ddot{\theta })\cos^{2}\phi -(\dot{\alpha }+\dot{\theta })\dot{\phi }\sin2\phi ]+2m*l\dot{l}(\dot{\alpha }+\dot{\theta })\cos^{2}\phi \\ +\dot{m}*l^{2}(\dot{\alpha }+\dot{\theta })\cos^{2}\phi +\frac{3\mu m*l^{2}}{2R^{3}}\sin2\theta \cos^{2}\phi =Q_{\theta } \end{array}$$

(12) $$\dot{m}*l^{2}\dot{\phi }+2m*l\dot{l}\dot{\phi }+m*l^{2}\ddot{\phi }+\frac{1}{2}m*l^{2}{\left(\dot{\alpha }+\dot{\theta }\right)}^{2}\sin2\phi +\frac{3\mu m*l^{2}}{2R^{3}}\sin2\phi \cos^{2}\theta =Q_{\phi }$$

(13) $$\begin{array}{l} {\dot{m}}^{\#}\dot{l}+m^{\#}\ddot{l}-\frac{1}{2}(m*)’l^{2}[{\dot{\phi }}^{2}+{\left(\dot{\alpha }+\dot{\theta }\right)}^{2}\cos^{2}\phi ]-m*l[{\dot{\phi }}^{2}+{\left(\dot{\alpha }+\dot{\theta }\right)}^{2}\cos^{2}\phi ]\\ -\frac{1}{2}(m^{\#})\prime {\dot{l}}^{2}+\frac{\mu (m*)\prime l^{2}}{2R^{3}}(1-3\cos^{2}\theta \cos^{2}\phi )+\frac{\mu m*l}{R^{3}}(1-3\cos^{2}\theta \cos^{2}\phi )=Q_{l} \end{array}$$

where $(\;{)}^{\prime }=d(\;)/dl$, $\;m*=m_{t}(3m_{1}-3m_{2}-m)/(6m)$, $m^{\#}=m_{t}(2m_{1}-m)/m$, $Q_{l}=-T$ is the tension control and the generalized forces, $Q_{\theta }$ and $Q_{\phi }$, are typically assumed to be negligible as a result of distributed forces along the tether. It should be noted that tether length can be controlled by deployment/retrieval of the winch control mechanism in the mother satellite; therefore, $m_{1},\;m_{t}$ in Equations (9)–(13) are functions of tether length.

Based on the premise of a Keplerian reference orbit for the center of mass as the independent variable, the orbit’s true anomaly, ν, is used to replace the generalized coordinate, α, in order to withdraw the premise, which can be expressed as follows:

(14) $$\dot{\nu }=\sqrt{\frac{\mu_{e}}{a^{3}{\left(1-e^{2}\right)}^{3}}}\kappa^{2},\;R=\frac{a(1-e^{2})}{\kappa }$$

where e is the orbital eccentricity, and a is the semi-major axis of the orbit, κ=1+ecosν. $\frac{d(i)}{dt}=\frac{d(i)}{d\nu }\times \frac{d\nu }{dt}\Rightarrow \dot{i}=i^{\prime }\dot{\nu },\;\ddot{i}=i^{\prime \prime }{\dot{\nu }}^{2}+\frac{\ddot{\nu }i^{\prime }}{\dot{\nu }},\;i=\theta ,\;\phi ,\;l$ is utilized by Equations (9)–(13). Nondimensional equations of motion can be written as follows:

(15) $$\theta^{\prime \prime }=2(\theta^{\prime }+1)[\frac{e\sin\nu }{\kappa }+\phi^{\prime }\tan\phi -\Phi_{1}\frac{\Lambda^{\prime }}{\Lambda }]-\frac{3}{2\kappa }\sin2\theta$$

(16) $$\phi^{\prime \prime }=\frac{2e\sin\nu }{\kappa }\phi^{\prime }-2\Phi_{1}\frac{\Lambda^{\prime }}{\Lambda }\phi^{\prime }-\frac{1}{2}[{\left(\theta^{\prime }+1\right)}^{2}+\frac{3}{\kappa }\cos^{2}\theta ]\sin2\phi$$

(17) $$\begin{array}{l} \Lambda^{\prime \prime }=\frac{2e\sin\nu }{\kappa }\Lambda^{\prime }-\Phi_{2}\frac{{\Lambda^{\prime }}^{2}}{\Lambda }+\Phi_{3}\Lambda [{\phi^{\prime }}^{2}+{\left(\theta^{\prime }+1\right)}^{2}\cos^{2}\phi \\ \;\;\;\;\;\;\;+\frac{1}{\kappa }(3\cos^{2}\theta \cos^{2}\phi -1)]-\frac{mT}{m_{1}{\dot{\nu }}^{2}L(m_{2}+m_{t})} \end{array}$$

where $(\;{)}^{\prime }=d(\;)/d\nu$, Λ=l/L is the nondimensional tether length, and L is the reference tether length. $\Phi_{i},\;i=1,\;2,\;3$ is the nondimensional coefficient.

(18) $$\Phi_{1}=\frac{m_{1}(m_{2}+m_{t}/2)}{mm*},\;\Phi_{2}=\frac{(2m_{1}-m)m_{t}}{2m_{1}(m_{2}+m_{t})},\;\Phi_{3}=\frac{m_{2}+m_{t}/2}{m_{2}+m_{t}}$$

## 3. Dynamic Analysis of Simplified Models of Single-DOF and Two-DOFs

In order to clarify dynamic behavior mechanisms of TSS and to explore the influence of various parameters on dynamic responses, Equations (15)–(17) need to be simplified, assuming that the tether length is fixed when system configurations remain fixed.

### 3.1. Single-DOF (θ)

Numerous studies show that tether in-plane angles are much larger than out-of-plane angles; when TSSs are operating in orbital planes, ϕ=0. In this case, Equations (15)–(17) can be rewritten as follows:

(19) $$\theta^{\prime \prime }=2(\theta^{\prime }+1)\frac{e\sin\nu }{\kappa }-\frac{3}{\kappa }\sin\theta \cos\theta$$

assuming that a spherical earth is considered. Orbital eccentricity, e, is a small quantity; thus, the perturbation method was selected in order to calculate approximate analytical solutions for Equation (19), where e is regarded as a tiny perturbation that is substituted into Equation (19).

(20) $$\theta^{\prime \prime }-2(\theta^{\prime }+1)\frac{e\sin\nu }{1+e\cos\nu }+\frac{3\sin2\theta }{2(1+e\cos\nu )}=0$$

The power series form of the periodic solution can be written as follows.

(21) $$\theta_{p}(\nu ,\;e)=e\cdot \theta_{1}(\nu )+e^{2}\cdot \theta_{2}(\nu )+e^{3}\cdot \theta_{3}(\nu )+e^{4}\cdot \theta_{4}(\nu )+e^{5}\cdot \theta_{5}(\nu )$$

The linear ordinary differential equation is written as follows.

(22) $$\begin{array}{l} {\theta^{\prime \prime }}_{1}+3\theta_{1}=2\sin\nu \\ {\theta^{\prime \prime }}_{2}+3\theta_{2}=2{\theta^{\prime }}_{1}\sin\nu -{\theta^{\prime \prime }}_{1}\cos\nu ,\;{\theta^{\prime \prime }}_{3}+3\theta_{3}=2{\theta^{\prime }}_{2}\sin\nu -{\theta^{\prime \prime }}_{2}\cos\nu \\ {\theta^{\prime \prime }}_{4}+3\theta_{4}=2{\theta^{\prime }}_{3}\sin\nu -{\theta^{\prime \prime }}_{3}\cos\nu ,\;{\theta^{\prime \prime }}_{5}+3\theta_{5}=2{\theta^{\prime }}_{4}\sin\nu -{\theta^{\prime \prime }}_{4}\cos\nu \end{array}$$

Equation (22) can be executed by the periodic initial condition, $\theta_{i}(0,\;\theta_{i0})=\theta_{i}(2\pi ,\;\theta_{i0})$i=1, 2,…,5, and the analytical solution of Equation (19) can be expressed as follows.

(23) $$\begin{array}{l} \theta_{p}=e\sin\nu -\frac{3}{2}e^{2}\sin2\nu +e^{3}\sin3\nu -\frac{3}{26}e^{4}(5\sin4\nu +13\sin2\nu )\\ \;\;\;\;\;\;+\frac{3}{143}e^{5}(143\sin\nu +55\sin3\nu -5\sin5\nu ) \end{array}$$

**Figure 2:**
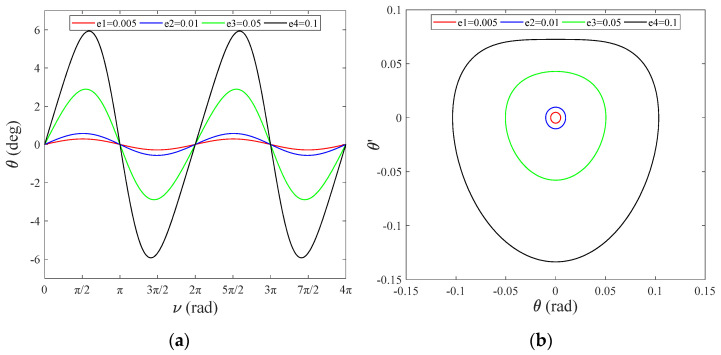
(**a**) Tether in-plane angle, θ, versus ν; (**b**) angular velocity of the in-plane angle, $\theta^{\prime }$, versus θ.

Figure 2a shows the tether in-plane vibration angle versus the orbit’s true anomaly expressed in radians with various e. The system moves periodically and repeatedly with a period of 2π in the direction of θ, and $e=0.1,\;\theta_{\max}=5.93{}^{\circ}$ appears at 1/4 and 3/4 of the period, respectively. Figure 2b illustrates that $\theta_{\max},\;\theta_{\max}^{\prime }$ increase as orbital eccentricity increases e→1 (the elliptical orbit is flatter), which means that the system tends to move towards an unstable equilibrium state.

### 3.2. Two-DOFs (θ,ϕ)

The tether out-of-plane vibration angle, ϕ, is considered based on a simplified single-DOF model. In this case, Equations (15)–(17) can be rewritten as follows.

(24) $$\theta^{\prime \prime }=2(\theta^{\prime }+1)[\frac{e\sin\nu }{\kappa }+\phi^{\prime }\tan\phi ]-\frac{3}{2\kappa }\sin2\theta$$

(25) $$\phi^{\prime \prime }=\frac{2e\sin\nu }{\kappa }\phi^{\prime }-[{\left(\theta^{\prime }+1\right)}^{2}+\frac{3}{\kappa }\cos^{2}\theta ]\frac{1}{2}\sin2\phi$$

**Figure 3:**
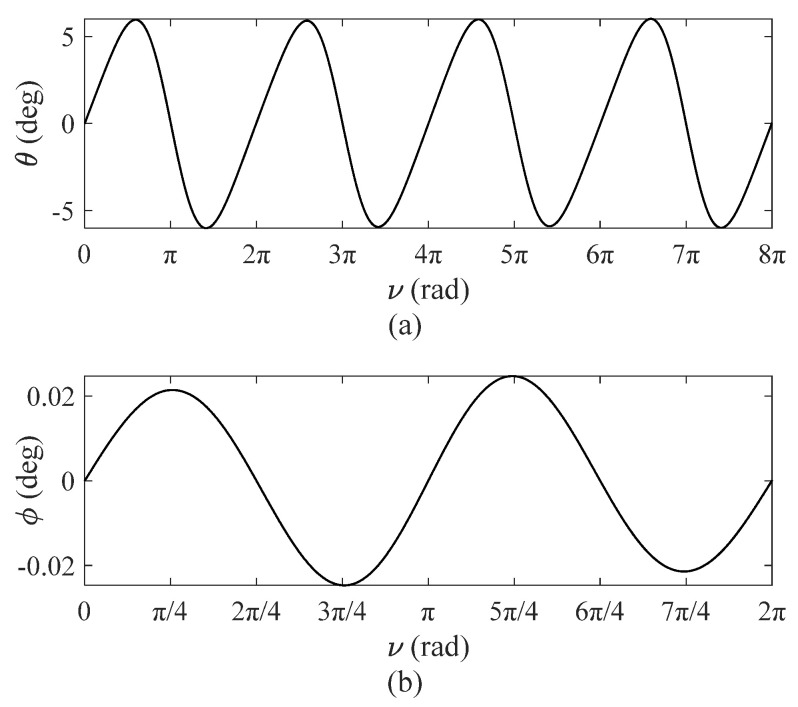
Tether vibration angles, *e* = 0.1: (**a**) tether in-plane angles versus *ν*; (**b**) tether out-plane vibration angles versus *ν*.

Figure 3 shows tether in-plane and out-of-plane vibration angles, θ, ϕ, versus ν.

In Equations (24) and (25), orbital eccentricity is 0.1, and initial values of the single-DOF motion solution $\theta_{0},\;{\theta^{\prime }}_{0}$ are adopted by Equation (24). As shown in Figure 3, numerical simulations show that the out-of-plane vibration angle is relatively small, and the maximum of ϕ is 0.0247°, far less than the in-plane angle, which has a slight effect on TSS dynamic response. Hence, the effect of the out-of-plane angle is negligible. However, coupling errors caused by out-of-plane vibrations to in-plane vibrations still require further numerical verification.

**Figure 4:**
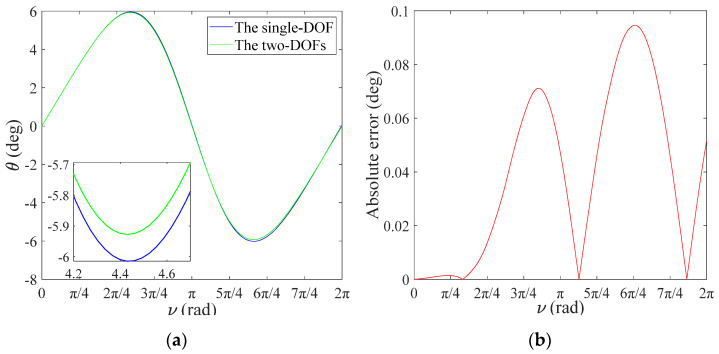
Coupling effects of ϕ, e=0.1: (**a**) numerical comparison for θ; (**b**) absolute error for θ.

**Figure 5:**
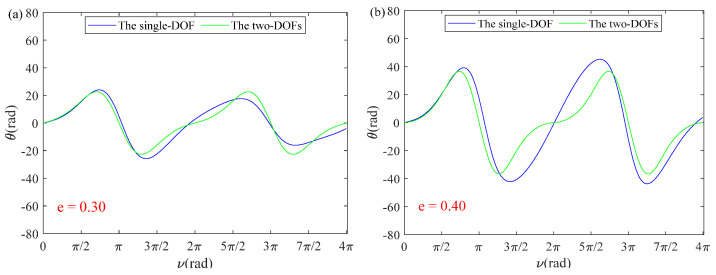

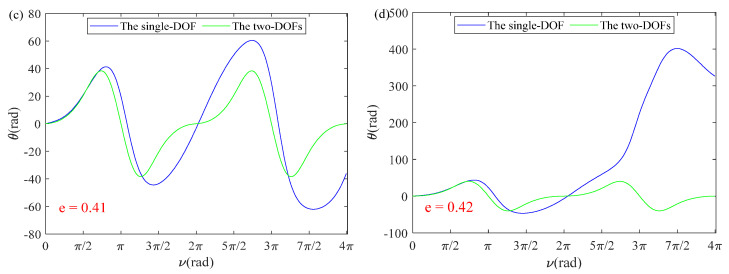
Coupling effects of *ϕ*: (**a**) *e* = 0.30; (**b**) *e* = 0.40; (**c**) *e* = 0.41; (**d**) *e* = 0.42.

In order to further verify the accuracy of the single-DOF simplified model, different orbital eccentricity values are substituted into Equations (24) and (25). Both curves almost coincide in Figure 4a, and the maximum error of the in-plane vibration angle is 0.0945° with e=0.1 in Figure 4b, which illustrates that coupling effects of the out-of-plane vibration angle are negligible. In particular, the solution of the single-DOF with the first five orders demonstrates sufficient accuracy in Equation (23), which proves that the error of the perturbation method is negligible. However, as shown in Figure 5, it can be easily observed that the error of the single-DOF simplified model increases as orbital eccentricity increases. In Figure 5, the error of the single-DOF simplified model was significantly smaller when value e decreased from 0.42 to 0.1, which means that a single DOF-simplified model can be applied to orbits with low orbital eccentricity so that accuracy can be guaranteed.

**Remark** **1.**  *The accuracy of the simplified model of TSS is strongly influenced by orbital eccentricity. For low-eccentricity orbits, a simplified model of TSS can significantly reduce calculation time.*

## 4. Stable Deployment Laws of Tether Release Rate and Tether Tension Control

Entire TSS configurations require transformation according to different mission requirements. Changing the tether length is the most direct control method of system conception transformation, which includes tether release rate control and tether tension control. During TSS transformation, tether release rate and tether tension control parameters are controlled by a deployment device in the mother satellite.

### 4.1. Tether Release Rate Control

The tether release rate is directly controlled by a winch control mechanism in the mother satellite, and the influences of tether tension and ϕ are ignored. Hence, Equations (15)–(17) can be rewritten as follows:

(26) $$\theta^{\prime \prime }=2(\theta^{\prime }+1)[\frac{e\sin\nu }{\kappa }-\frac{l^{\prime }}{l}]-\frac{3}{2\kappa }\sin2\theta$$

where $l^{\prime }/l$ is the pseudo damping term, which makes θ, ϕ convergent.

#### 4.1.1. Fixed Angle θ

The system’s in-plane angle is assumed to be a fixed angle of nonrotating motion. Corresponding to actual conditions, a Global Positioning System (GPS) rotates around the earth at a fixed angle in order to produce a stable state. $\theta =\theta_{0},\;\theta^{\prime }=\theta^{\prime \prime }=0$ are substituted into Equation (26), which can be written as follows.

(27) $$\frac{l^{\prime }(\nu )}{l(\nu )}=\frac{e\sin\nu }{1+e\cos\nu }-\frac{3\sin2\theta_{0}}{4(1+e\cos\nu )}$$

The log ratio of tether length, $\ln\left[l(\nu )/l_{0}(\nu )\right]$, can be obtained by integrating Equation (27).

**Figure 6:**
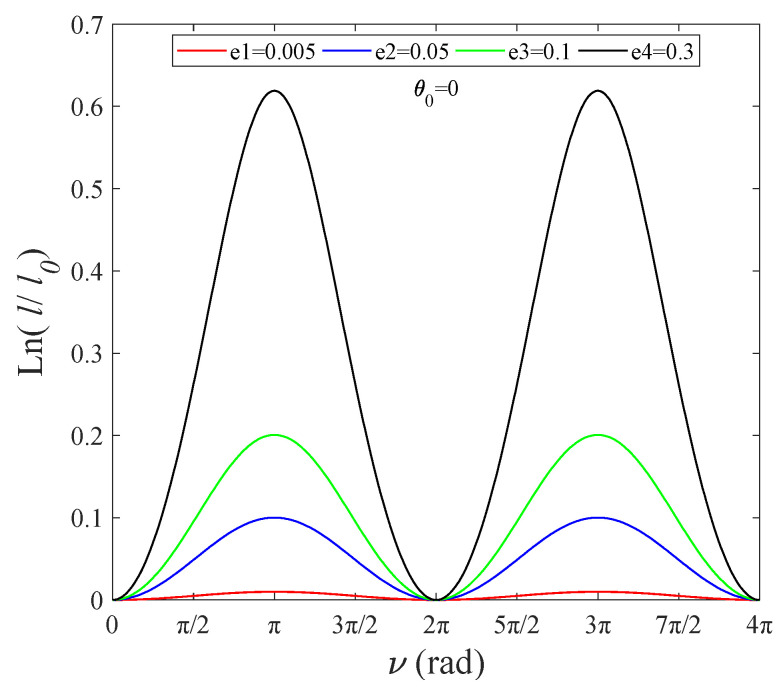
Log ratio of tether length versus ν, θ=const.

Figure 6 shows that Equation (27) achieves a unified analytic solution when $\theta_{0}=k\pi /2$ (*k* is an integer). Tether length is positively related to orbital eccentricity, and the abscissa corresponding to the highest point is ν=(2k+1)π.

#### 4.1.2. Fixed Angular Velocity $\theta^{\prime }$

The system is assumed to rotate steadily and uniformly in the direction of the in-plane angle, $\theta =\omega \nu +\theta_{0}$, where ω is the angular velocity, and $\theta_{0}$ is the initial value. θ’=ω and θ’’=0 are substituted into Equation (26), which can be written as follows.

(28) $$\frac{l^{\prime }(\nu )}{l(\nu )}=\frac{e\sin\nu }{1+e\cos\nu }-\frac{3\sin[2(\omega \nu +\theta_{0})]}{4(\omega +1)(1+e\cos\nu )}$$

The log ratio of tether length can be obtained by applying integration.

**Figure 7:**
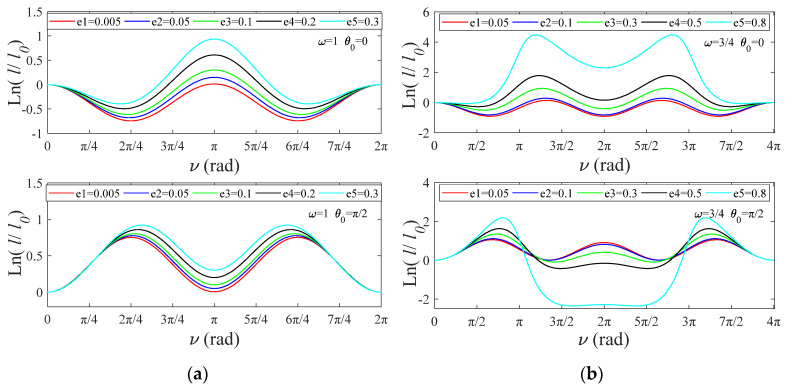
Log ratio of tether length versus ν, $\theta^{\prime }=const$: (**a**) $\omega =1,\;\theta_{0}=0\;\mathrm{and}\;\pi /2$; (**b**) $\omega =3/4,\;\theta_{0}=0\;\mathrm{and}\;\pi /2$.

As shown in Figure 7, tether length amplitude is positively related to e, and the system requires longer tethers to achieve stability control. The results above can be used to guide TSS system attitude control. When tether release conditions satisfy Equation (28), TSS systems can operate at a fixed angular velocity, $\theta^{\prime }$.

### 4.2. Tether Tension Control

In order to enhance efficiency, applicability and stability, tether tension control is facilitated by TSS [34]. It is assumed that tether tension is the same at every point of the tether and is equal to the tension at the point of deployment/retrieval. The tether braking mechanism is modeled after the SEDS deployer, which uses a friction brake in order to control tether deployment speed [35]. Tether tension is expressed as follows:

(29) $$T=\left[T_{0}+I\rho {\dot{l}}^{2}{\left(1-A_{\mathrm{sol}}l/L_{\mathrm{ref}}\right)}^{-E}\right]\exp\left(f_{\theta }\left|\theta -\theta_{0}\right|+2\pi f_{n}n*\right)$$

where $\dot{l}$ is the tether release rate. Tether tension control parameters are listed in Table 1.

A numerical simulation was performed in order to demonstrate control law performance, and simulation parameters are listed in Table 2, where $\mu_{e}=398,600\;{\mathrm{km}}^{3}{/s}^{2}$ is Earth’s gravitational coefficient.

**Figure 8:**
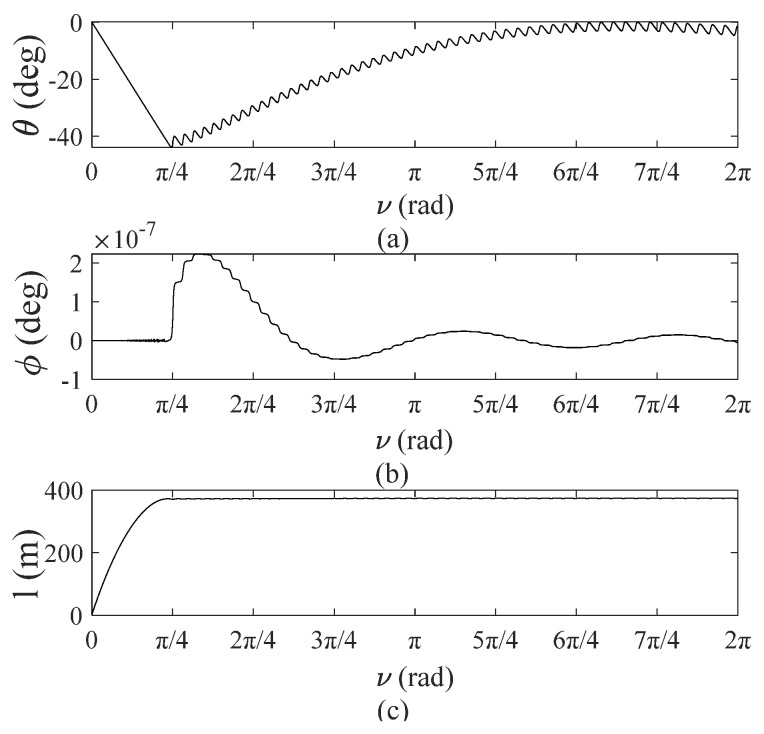
Deployment process of TSS: (**a**) tether in-plane angles versus *ν*; (**b**) tether out-plane vibration angles versus *ν*; (**c**) tether length versus *ν*.

Figure 8 shows the dynamic response of the TSS deployment process. Figure 8a,b show variations of in-plane and out-of-plane pitch angles and roll angles versus the true anomaly, ν. It can be concluded that the system approaches the expected angle, 0 rad, after swinging under an initial perturbation. This result illustrates that a controlled deployment process is asymptotically stable and demonstrates the validity of the tether tension control equation (Equation (29)). Figure 8c shows tether length during deployment, which exhibits a smooth deployment curve, and the tether eventually reaches a stable length.

**Remark** **2.**  *Analysis results of tether release rate control and tether tension control laws can provide effective feedback for TSS position and attitude.*

## 5. Stability Analysis of TSS Deployment Using Floquet Theory

Floquet theory is used to analyze the stability of solutions of linear ordinary differential equations with periodic variable coefficients. Local stability of deployment along preassigned pitch angles and roll angles can be analyzed by using Floquet theory. Tether length remains unchanged once the non-dimensional tether length equals one after accomplishing deployment in Section 4.2. Steps of Floquet theory applied to TSS systems are shown as follows.

In the case of $p_{1}=\theta ,\;p_{2}=\theta^{\prime },\;p_{3}=\phi ,\;p_{4}=\phi^{\prime }$, the matrix form of Equations (15)–(17) can be summarized as follows:

(30) $$P=\left[\begin{array}{c} {p^{\prime }}_{1}\\ {p^{\prime }}_{2}\\ {p^{\prime }}_{3}\\ {p^{\prime }}_{4} \end{array}\right]=\left[\begin{array}{c} \theta^{\prime }\\ 2(\theta^{\prime }+1)(\frac{e\sin\nu }{\kappa }+\phi^{\prime }\tan\phi )-\frac{3}{2\kappa }\sin(2\theta )\\ \phi^{\prime }\\ \frac{2e\phi^{\prime }\sin\nu }{\kappa }-\frac{1}{2}[{\left(\theta^{\prime }+1\right)}^{2}+\frac{3}{\kappa }\cos^{2}\theta ]\sin(2\phi ) \end{array}\right]$$

where $p={\left(\theta ,\;\theta^{\prime },\;\phi ,\;\phi^{\prime }\right)}^{T}$ are state-space vectors, and $p_{s}={\left(\theta_{s},\;{\theta^{\prime }}_{s},\;\phi_{s},\;{\phi^{\prime }}_{s}\right)}^{T}$ are equilibrium points, ${\theta^{\prime }}_{s}=\frac{d\theta_{s}}{d\nu }$. Equation (30) can be expressed as follows:

(31) $$\Phi \prime =A(p_{s})\Phi$$

where $A(p_{s})$ is the Jacobian matrix of vector function, P, in a small neighborhood near the equilibrium point, $p_{s}$.

(32) $$A(p_{s})=\left[\begin{array}{cccc} 0 & 1 & 0 & 0\\ -\frac{3}{\kappa }\cos(2p_{1s}) & 2(\frac{e\sin\nu }{\kappa }+p_{4s}\tan p_{3s}) & \frac{2p_{4s}(p_{2s}+1)}{\cos^{2}p_{3s}} & 2(p_{2s}+1)\tan p_{3s}\\ 0 & 0 & 0 & 1\\ \frac{3}{2\kappa }\sin(2p_{1s})\sin(2p_{3s}) & -(p_{2s}+1)\sin(2p_{3s}) & -[{\left(p_{2s}+1\right)}^{2}+\frac{3}{\kappa }\cos^{2}p_{1s}]\cos(2p_{3s}) & \frac{2e\sin\nu }{\kappa } \end{array}\right]$$

The period is 2π, and it can be expressed as follows.

(33) A(ν, e)=A(ν+2π, e)

The monodromy matrix can be obtained by integrating Equation (32) for one period from initial time ν=0, which combines with the initial condition $\Phi (\nu_{0},\;e)=I$, where $I_{4\times 4}$ is the identity matrix.

(34) $$M=\Phi (2\pi ,\;e)=e^{\int_{0}^{2\pi }A(\nu )d\nu }$$

According to Floquet theory, the stability of the zero solution of Equations (15)–(17) can be assessed by a Floquet multiplier:

(35) $$\left\{ \begin{array}{l} {\left|\lambda_{i}\right|}_{\max}<1,\;(i=1,\;2,\;3,\;4)\;\;\;\mathrm{Asymptotically}\;\mathrm{stable}\\ {\left|\lambda_{i}\right|}_{\max}=1,\;(i=1,\;2,\;3,\;4)\;\;\;\mathrm{Undetermined}\\ {\left|\lambda_{i}\right|}_{\max}>1,\;(i=1,\;2,\;3,\;4)\;\;\;\mathrm{Unstable} \end{array}\right.$$

where ${\left|\lambda_{i}\right|}_{\max}$ is a Floquet multiplier that is the maximum of the absolute value of $\lambda_{i}$, and λ is an eigenvalue of the monodromy matrix, M, in Equation (34).

**Figure 9:**
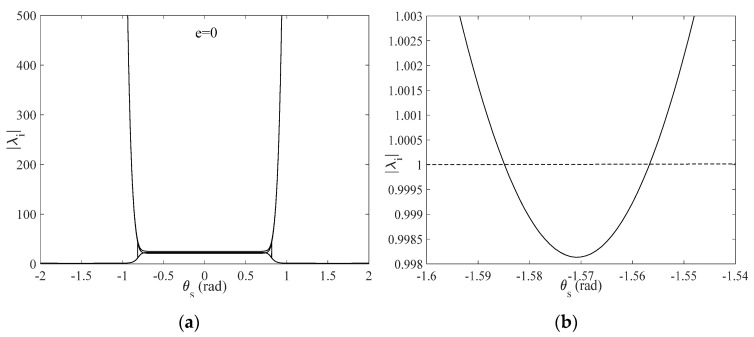
(**a**) Floquet multipliers versus $\theta_{s}$, e=0; (**b**) local enlargement of Floquet multipliers versus $\theta_{s}$.

Figure 9a shows the relationship between Floquet multipliers and stability of expected in-plane angles for e=0, where the case of $\phi_{s}=0$ is discussed. As shown in Figure 9b, this result shows that Floquet multipliers are less than one for $\theta_{s}\in (-1.584,\;-1.563)\;\mathrm{and}\;(1.563,\;1.584)$ by symmetry, which illustrates that deployment in a short interval of $\theta_{s}$ is asymptotically stable. However, once $\theta_{s}$ lies outside the specified range, Floquet multipliers are always greater than one, which shows that the deployment process is undetermined since the expected in-plane angle lies outside the domain of stability. A similar result is achieved with e∈(0, 0.5).

## 6. Conclusions

In this paper, nonlinear dynamic characteristics of TSS during a configuration conversion process were analyzed based on a simplified rigid-rod model. Tether tension control was proposed, and numerical simulations show that the proposed law can suppress in-plane and out-of-plane librations of rigid tethered satellites during deployment, and spacecraft and tether stability control goals can be achieved. The periodic stability of time-varying control systems was analyzed by using Floquet theory, and small parameter regions of TSS in asymptotically stable states were expressed.

In summary, this paper has provided tether release rate and tether tension control laws for suppressing wide-ranging TSS vibrations that are valuable for improving TSS attitude control accuracy and performance, specifically for TSSs that are operating in low-eccentricity orbits. Additionally, future studies based on existing research can be conducted with respect to two aspects: (1) A more accurate model can be established since, in the current study, the tether was discretized into a series of lumped masses connected by springs and dampers with mass. (2) Applications of accurate models can generate more dimensions, and Floquet theory used to analyze the stability of high-dimensional dynamic systems requires further verification.

## Figures and Tables

**Table 1:** Tether tension control law.

Parameters	Value
Minimal tension as a result of friction, *T*_0_	0.01 N
Inertial multiplier, *I*	3.1
$\mathrm{Tether}\;\mathrm{annulus}\;\mathrm{solidity},\;A_{\mathrm{sol}}$	0.89
Area exponent, *E*	1.4
$\mathrm{Friction}\;\mathrm{coefficient}\;\mathrm{over}\;\mathrm{the}\;\mathrm{exit}\;\mathrm{guide},\;f_{\theta }$	0.18
$\mathrm{Zero}\;\mathrm{friction}\;\mathrm{exit}\;\mathrm{angle},\;\theta_{0}$	0
$\mathrm{Friction}\;\mathrm{coefficient}\;\mathrm{over}\;\mathrm{the}\;\mathrm{brake}\;\mathrm{pole},\;f_{n}$	0.05
Number of effective brake turns of the tether, n*	1.9

**Table 2:** TSS parameter values.

Parameters	Value
Mother satellite mass, *m*_1_	6530 kg
Subsatellite mass, *m*_2_	12 kg
Tether diameter, *d*	5 × 10^−4^ m
Reference tether length, *L*	3500 m
Tether line density, *ρ*	1.85 × 10^−4^ kg/m
Orbit eccentricity, *e*	0.0027
Orbital semi-major axis, *a*	6.645 × 10^6^ m
$\mathrm{Earth}’s\;\mathrm{gravitational}\;\mathrm{coefficient},\;\mu_{e}$	3.986 × 10^14^ m^3^/s^2^

## Data Availability

All necessary and relevant data are included in this paper.
